# Mortality-Risk Prediction Model from Road-Traffic Injury in Drunk Drivers: Machine Learning Approach

**DOI:** 10.3390/ijerph181910540

**Published:** 2021-10-08

**Authors:** Wachiranun Sirikul, Nida Buawangpong, Ratana Sapbamrer, Penprapa Siviroj

**Affiliations:** 1 Department of Community Medicine, Faculty of Medicine, Chiang Mai University, Chiang Mai 50200, Thailand; wachiranun.sir@cmu.ac.th (W.S.); lekratana56@yahoo.com (R.S.); 2 Center of Data Analytics and Knowledge Synthesis for Health Care, Chiang Mai University, Chiang Mai 50200, Thailand; 3 Department of Family Medicine, Faculty of Medicine, Chiang Mai University, Chiang Mai 50200, Thailand; nidalooknum@gmail.com

**Keywords:** alcohol, drunk driver, road-traffic injury, machine learning

## Abstract

Background: Alcohol-related road-traffic injury is the leading cause of premature death in middle- and lower-income countries, including Thailand. Applying machine-learning algorithms can improve the effectiveness of driver-impairment screening strategies by legal limits. Methods: Using 4794 RTI drivers from secondary cross-sectional data from the Thai Governmental Road Safety Evaluation project in 2002–2004, the machine-learning models (Gradient Boosting Classifier: GBC, Multi-Layers Perceptrons: MLP, Random Forest: RF, K-Nearest Neighbor: KNN) and a parsimonious logistic regression (Logit) were developed for predicting the mortality risk from road-traffic injury in drunk drivers. The predictors included alcohol concentration level in blood or breath, driver characteristics and environmental factors. Results: Of 4974 drivers in the derived dataset, 4365 (92%) were surviving drivers and 429 (8%) were dead drivers. The class imbalance was rebalanced by the Synthetic Minority Oversampling Technique (SMOTE) into a 1:1 ratio. All models obtained good-to-excellent discrimination performance. The AUC of GBC, RF, KNN, MLP, and Logit models were 0.95 (95% CI 0.90 to 1.00), 0.92 (95% CI 0.87 to 0.97), 0.86 (95% CI 0.83 to 0.89), 0.83 (95% CI 0.78 to 0.88), and 0.81 (95% CI 0.75 to 0.87), respectively. MLP and GBC also had a good model calibration, visualized by the calibration plot. Conclusions: Our machine-learning models can predict road-traffic mortality risk with good model discrimination and calibration. External validation using current data is recommended for future implementation.

## 1. Introduction

Road-traffic injury (RTI) is currently a major public health issue and a leading cause of mortality among all age groups, particularly children and young adults [1]. According to a WHO report, 93% of the world’s fatalities on the roads occur in low- and middle-income countries [2]. Thailand is a middle-income country and has remained in the top ten for road-traffic deaths for many years [3].

Drunk driving is a key behavioral risk factor for increased risk of fatality and serious disabilities [4,5,6]. Even with low blood-alcohol concentration (BAC) levels, alcohol can increase the severity of RTI [7,8]. Various strategies, both at a national and at an individual level, have been implemented and have reduced alcohol-related fatalities and injuries [9,10,11]. Those focused on individual behavior include the perceived threat of being arrested, legislative penalties, and RTI severity. However, only the perceived threat of being arrested has been shown to influence individual behavior in avoiding drunk driving, but not punitive measures [12]. Raised awareness of RTI consequences using mass media campaigns and social activities was also an effective strategy, particularly when combined with checkpoints [9,13]. However, increasing the number of checkpoints and intensive mass media campaigns are too costly, especially in low–middle income countries. Therefore, the current strategies might not be enough and may need additional efforts for better management of this problem.

Although the legal limit of blood-alcohol level is a good indicator for defining impaired drivers, the intensity of impairment and RTI severity are also influenced by driver characteristics and environmental factors [14,15,16]. Therefore, using these factors combined with alcohol data has the feasibility to develop a more personalized drunk-driver screening strategy.

Prediction models are widely used to predict health events and for screening high-risk individuals [17,18]. Machine learning (ML) has become a popular approach for prediction model development in health care [19]. The advantages of ML are the ability to analyze diverse data types and perform complex computational algorithms [20]. It requires specific data preprocessing, complex parameter tuning, and understanding of each ML algorithm. Several ML algorithms have been applied to predict the severity of road-traffic injuries. The study of Artificial Neural Networks and classical decision tree algorithms was developed using precrash factors and could predict the severity of non-alcohol-related traffic injuries with acceptable performance [21]. Another study using the Recurrent Neural Network (RNN), Multilayer Perceptron (MLP) and Bayesian Logistic Regression found that only the RNN provided good accuracy [22]. The study of the decision tree-based algorithm (Random Forest: RF), nonparametric learning method (K-Nearest Neighbor: KNN), and modified traditional statistical model (Regularized Logistic Regression Classifier: Logit) also reported promising results in predicting road-traffic severity [23]. Furthermore, a recent study using ML algorithms synergized with clustering techniques (e.g., Fuzzy C-Means-based Support Vector Machines and Neural Networks) also obtained good performance in terms of accuracy and F1 score [24]. Nevertheless, there is no current evidence on the use of ML algorithms to predict the risk of alcohol-related traffic mortality.

Using machine-learning models instead of a legal limit of alcohol concentration or fixed prediction rules/methods (e.g., regression models, decision trees) may provide a more flexible, effective, and personalized tool for identifying drunk drivers at risk of road-traffic mortality. The major advantage of machine learning is its continuous learning, in which the model algorithm is constantly modified in response to newly derived data. For future implementation, these machine learning models may be integrated with the present sobriety checkpoint screening method to provide a personalized drunk-driving screening strategy. Therefore, we conducted the development and internal validation of ML models using driver characteristics, environmental factors, and alcohol testing results to evaluate the performance of ML models in alcohol-related traffic mortality prediction.

## 2. Materials and Methods

### 2.1. Derivation Dataset (Thai Governmental Road Safety Evaluation Project from 2002–2004)

#### 2.1.1. Data Collection

This study used retrospective cross-sectional data from the Thai Governmental Road Safety Evaluation project conducted by the Thai Health Promotion Foundation evaluation team in response to road safety planning from 2002–2004. The road-traffic injury data were collected in 4 main geographical regions of Thailand and directly reported to the information center by field investigators who co-operated with nurses at emergency departments from 29 main provincial hospitals.

#### 2.1.2. Study Population

The development of the prediction model was performed based on 4794 records of drivers who received emergency care or were transferred or admitted to a secondary and tertiary hospital during the long weekend periods of 2003 (27 December 2002–2 January 2003), Thai New Year 2003 (11–18 April 2003), and New Year 2004 (29 December 2003–4 January 2004). All the patient identification data including hospital numbers and citizen ID were completely removed and it was not possible to track back by using other characteristic data in the derivation dataset. Ethical approval of this study was obtained from the Research Ethics Committee of Faculty of Medicine, Chiang Mai University, Thailand (COM-2563-07841).

#### 2.1.3. Predictors

Candidate predictors for modeling were selected based on the previous evidence from road-traffic injury studies. The retrieved predictors from a dataset included alcohol odor on breath, breathing alcohol concentration (BrAC) and BAC. The alcohol concentration level was measured in patients attending accident and emergency departments with road-traffic injuries by a nurse at the triage point, or by the officer who assessed victims at the scene. The BAC samples were gathered immediately at emergency departments from all suspected alcohol-related road-traffic injury patients according to legislative measures at that time, and were delivered to the toxicology lab of the same hospital within 24 h. The BAC results were reported to authorized officers before being directly sent to the information center. In addition, the demographic data, location, driving in an unfamiliar area, and time of the road-traffic accident, type of vehicle, and safety used, were collected by field investigators from all study sites. Categorical variables—place of accident and vehicle type—were modified by one-hot encoding into binary features. The time of the accident was categorized based on the period of sobriety checkpoint shifts, which were 8:01 a.m. to 4:00 p.m., 4:01 p.m. to 12:00 midnight, and 12:01 midnight to 8:00 a.m. The continuous predictors, including age and BAC, were normalized before being used in model development.

#### 2.1.4. Outcomes

Death from the road-traffic injury was the primary outcome from our predictive models, which was derived from the Thai Governmental Road Safety Evaluation project data. Death location from the derived dataset was death at the scene, death during transfer, death at the emergency room, and death in hospital. The outcomes were obtained from police officers and medical records by investigators at study sites.

### 2.2. Missing Data and Imputation

**Figure 1:**
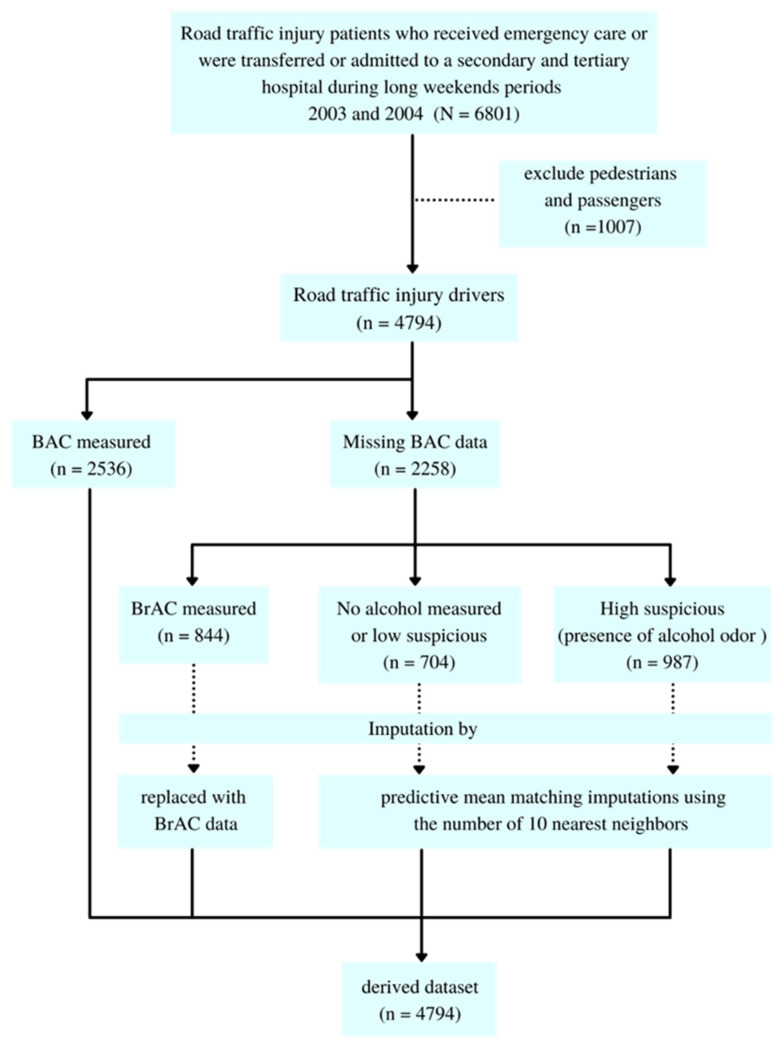
The flow diagram of derived data and missing data imputation.

The missing data were not found in the other candidate predictors except BAC. From 4794 records, 2536 (52.90%) had BAC missing data. 844 (17.61%) were missing because a breathalyzer was used instead of the BAC test. Hence, those values were replaced by BrAC results which is the relative measurement of BAC [25,26]. There were 705 records (14.71%) with BAC missing data due to a low suspicion of alcohol use, which was consistent with the absence of alcohol odor. The other missing data were found in 987 drivers (20.58%) whose alcohol odor was detected but no BAC or BrAC measure was performed. Therefore, we imputed these data by predictive mean matching imputations using the numbers of 10 nearest neighbors. The flow diagram of derived data and missing data imputation is displayed in Figure 1.

### 2.3. Model Development

The mortality prediction model was developed by the information of candidate variables. The derived models were developed using two datasets, which were the imbalanced dataset and the rebalanced dataset. The minority class of the imbalanced dataset was oversampled into a 1:1 ratio (4365 survivors: 4365 dead drivers) by the Synthetic Minority Oversampling method (SMOTE) to obtain the rebalanced dataset. Machine-learning algorithms using Python programming and the Sci-kit learn package were implemented in this study including the K-Nearest Neighbors (KNN) algorithm, ensemble tree-based ML algorithms: Random Forest classifier (RF), Stochastic Gradient Boosting Classifier (GBC), Multilayer Perceptron Artificial Neural Network (MLP) and Logistic Regression model. The details of machine-learning algorithms and their hyperparameters are described below. The feasible predictors were obtained using stepwise variable choosing with backward elimination based on a significant threshold of *p*-value < 0.100 by Multi-variate Logistic Regression. ML hyperparameters were determined by using a grid search with 10-fold cross-validation (GridSearchCV) on the derived dataset to determine the parameters that led to the best performance. For the GridSearchCV function, the dictionary of model hyperparameters or “parameter grid” is defined based on the model preferences as described below. This function performed hyperparameter optimization by exhaustively searching for the best parameters from all combinations of values in parameter grids and also performed k-fold cross-validation to estimate the performance score.

#### 2.3.1. K-Nearest Neighbors (KNN)

KNN, or neighbor-based classification, is an instance-based or nongeneralizing learning approach. Classification is determined by a simple majority vote of each point’s nearest neighbors: a query point is allocated to the data class having the most representation among its nearest neighbors. The hyperparameters in KNN are the number of neighbors (K), weight function (“Uniform weights” assigns equal weights to all points or “Distance weights” points by the inverse of their distance to a query point), the method of distance measurement (e.g., Euclidean method or Manhattan method), and the algorithm used to compute the nearest neighbors including Auto, Ball tree, K-D tree, and Brute-force searches.

#### 2.3.2. Random Forest Classifier (RF)

The RF classifier is an ensemble decision-tree based method, which eradicates the limitations of a classic decision-tree algorithm, including overfitting of datasets, and increases discrimination performance. The RF algorithm is based on a variety of decision trees, which are generated by bootstrap sampling and selected variables. Each decision tree is composed of three types of nodes: decision nodes, leaf nodes, and a root node. Each tree’s leaf node represents the decision tree’s final results, which is determined using a majority-voting mechanism. The main parameters to optimize when using this method are the number of trees in the forest and the maximum features in each tree. The other hyperparameters in RF are the maximum depth of the tree and the number of nodes.”

#### 2.3.3. Stochastic Gradient Boosting Classifier (GBC)

GBC is a group of combined weak learning models that generate more effective machine-learning models. The core principle of GBC is based on the boosting method (e.g., AdaBoost), which is to fit a sequence of weak learners (e.g., models that are only slightly better than random guessing, such as small decision trees) to make a classification on repeatedly modified versions of the data. This algorithm weights the input observations in the training set, providing a higher sample weight to observations that are difficult to classify. Additional weak learners are sequentially added to the algorithms and assigned to the most difficult classified observations. The predictions are made through majority vote, with the observations being classified according to which class receives the most votes from the weak learners. Gradient boosting classifiers combine the boosting method with error minimization using loss functions to minimize the prediction error between the actual and the predicted classes. The hyperparameters of GBC in generating the boosting algorithm are the number of weak learners, the maximum depth of decision trees, and the maximum features in each tree. The learning rate is a hyperparameter in the range 0 to 1 that controls weight applied to each weak learner at each boosting iteration. For error minimization, the loss functions (e.g., binomial deviance (provides probability estimates) and exponential loss) can be specified by hyperparameter tuning.

#### 2.3.4. Multilayer Perceptron Artificial Neural Network (MLP)

MLP is a supervised learning algorithm using the concept of a neural network. The simplest elements of MLP are the input layer, the hidden layer, and the output layer of the perceptron/neuron. There are basically three steps in the training of the MLP model. The input data is entered via the input layer, passes through the hidden layers to the output layer to obtain the predicted class, and calculates the error by a specified loss function. Then, the calculated error will be backpropagated to optimize the weights and bias of each perceptron to minimize the prediction error. To make a precise classification, the hidden layer sizes, the activation functions of the hidden layers (e.g., ReLU, Logistic, Identity), and the solver for weight optimization (e.g., Stochastic Gradient Descent, Quasi-Newton method) are required to be optimized by hyperparameter tuning. The other hyperparameters for the learning effectiveness are the learning rate (learning methods and initial learning rate) and the maximum number of training iterations.

#### 2.3.5. Logistic Regression Model (Logit)

Logistic regression model is also known in the literature as logit regression, maximum-entropy classification, or the log-linear classifier. In this approach, the target classification probabilities are modeled using a logistic function. Model transparency and interpretability is the major advantage of this approach. In addition, using the regularization approaches, which are an extension of the logit model, can improve model performance and decrease overfitting. The regularization techniques (e.g., L1, L2, Elastic-net), C-value (inverse of regularization strength; smaller values specify stronger regularization), and solver algorithms (e.g., Newton-CG, lbfgs, liblinear, sag, and saga) are the hyperparameters of the logit model.

### 2.4. Internal Validation, Discrimination Performance and Calibration

A 10-fold cross-validation method was performed for assessing model optimism and internal validation. The derived dataset is divided into 10 folds of data and repeated 10 times to perform model training and testing. For each iteration, nine folds of data are used to train the model and then tested with the remaining fold to ensure that almost all of the derived data were used to train and test the models. We assessed the discrimination performance by computing the area under the receiver operating characteristic curve (AUC) for each model. Estimates of discrimination performance were reported as the mean AUC across all repetitions of cross-validation. To further explain model performance, we also calculated secondary metrics of the models, including likelihood ratio, predictive values, specificity, and sensitivity. The equations for the secondary metrics calculation are provided below. The model calibration revealed the agreement between the observed proportion of classified outcomes and predicted probability from derived models. The calibration plot contrasted how well the probabilistic predictions of different classifiers were calibrated.

Sensitivity equation:

(1) $$Sensitivity=\frac{True\;positive}{True\;positive+False\;negative}$$

Specificity equation:

(2) $$Specificity=\frac{True\;negative}{True\;negative+False\;positive}$$

Positive predictive value (*PPV*) equation:

(3) $$PPV=\frac{True\;positive}{True\;positive+False\;positive}$$

Negative predictive value (*NPV*) equation:

(4) $$NPV=\frac{True\;negative}{True\;negative+False\;negative}$$

Positive likelihood ratio (PLR) equation:

(5) $$PPV=\frac{sensitivity}{1-specificity}$$

Negative likelihood ratio (NLR) equation:

(6) $$NPV=\frac{1-sensitivity}{specificity}$$

### 2.5. Statistical Analysis

The associations between predictors and outcomes were identified by statistical tests consisting of correlation analysis, chi-squared test, *t*-test for parametric values, Rank-sum test for nonparametric values, and multivariate logistic regression for predictors selection. All statistical analyses and missing data imputation were performed using statistical software by the STATA software package (Stata Corp. 2019. Stata Statistical Software: Release 16. College Station, TX, USA: Stata Corp LLC.). The data preprocessing, modeling, and performance analysis was conducted using Python (Python Software Foundation) with the Pandas package and the Sci-kit learn package. Additional details on the machine-learning models were shown in Table S1 in the Supplementary Materials.

## 3. Results

### 3.1. Baseline Characteristics of Drivers

Of 4794 drivers, 429 (8.94%) died from the RTI. In our study, most of the injured drivers were teenagers and young adolescents. Driver age was slightly higher in the survivor group (30 years, IQR 6 vs. 26 years, IQR 19; *p* = 0.001). Most drivers were male, and males were significantly more highly represented in road-traffic deaths compared to survivors (393, 91.61%) vs. 3804, 87.15%); *p* = 0.008). BAC levels in the road-traffic death group (15.00, IQR 156.70) were higher than the surviving drivers (1.00, IQR 130.00; *p* = 0.051) after imputing the missing data. Motorcycles were the most common vehicle used in both groups and the proportion of motorcycles used was significantly higher in the survivor group compared to the dead group (91.38% vs. 88.11%; *p* = 0.038). The usage of helmets in the survivor group was also significantly higher than the dead group (17.25% vs. 10.05%; *p* = 0.001). Safety-belt usage was also higher in the survivor group (31.98% vs. 9.68%; *p* = 0.010). The majority of road-traffic injuries occurred between midnight and 8:00 a.m., where the RTI deaths were considerably higher in than in the RTI survivors (52.68% vs. 44.88%; *p* = 0.002). Most of the accidents occurred in rural areas. The percentage of RTI deaths in these areas were significantly higher than the RTI survivors (53.85% vs. 47.26%; *p* = 0.009), whereas the RTI deaths were lower than the RTI survivors in urban areas (11.66% vs. 18.83%; *p* < 0.001). In addition, road-traffic deaths caused by driving across provinces were significantly higher than RTI survivors (25.87% vs. 13.45%, *p* < 0.001). The detail of driver characteristics from the derived dataset is presented in Table 1.

### 3.2. Model Development

Data from 4794 drivers were used for model development. The association between candidate predictors and road-traffic death by univariate analysis, multivariable regression, and AUC were reported in Table 2. Feature selection for model development was selected by a backward elimination approach via multivariable logistic regression. It was found that wearing a motorcycle helmet and wearing a seat belt were associated with decreased road-traffic deaths. Whereas other candidate variables were related to increased road-traffic mortality risk. However, the discrimination performance by each predictor showed a failure of poor performance. These predictors from the original data were used for the ML development and measured the model performances. Nevertheless, the discrimination performances of imbalance learning classifiers obtained a poor performance because the classifiers intended to classify only the majority class (Accuracy paradox). Therefore, a rebalancing strategy by SMOTE was applied to counter this problem. The oversampling data were generated and rebalanced the minority group in a 1:1 ratio. Finally, the derived data included 4365 RTI dead drivers and 4365 surviving drivers, and all candidate predictors, as shown in Table 2, were used in model development. The performances of imbalance learning models are provided (Table S2, Figures S1 and S2 in the Supplementary Materials).

### 3.3. Discrimination Performance and Model Calibration

**Figure 2:**
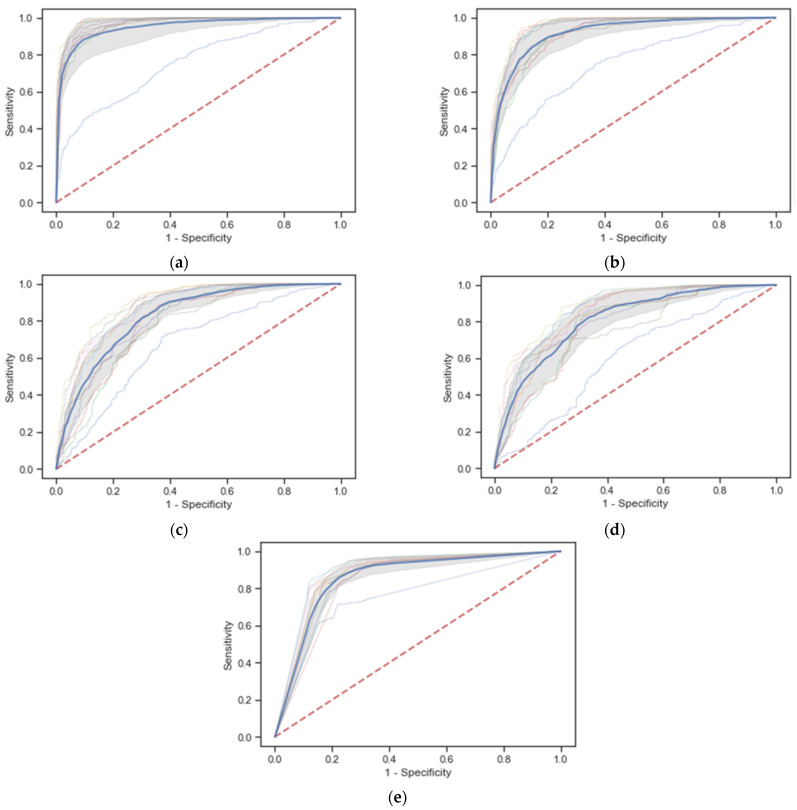
Receiver Operating Characteristic curves from 10-fold cross-validation of the rebalanced learning classifiers by SMOTE. (**a**) Gradient Boosting Classifier (GBC) model, mean AUC: 0.95, 95% CI: 0.90–1.00; (**b**) Random Forest (RF) model, mean AUC: 0.92, 95% CI: 0.87–0.97; (**c**) Multilayer Perceptron (MLP) model, mean AUC: 0.73, 95% CI: 0.78–0.88; (**d**) Regularized Logistic Regression (Logit) model, mean AUC: 0.73, 95% CI: 0.78–0.88; (**e**) K-Nearest Neighbor (KNN) model, mean AUC: 0.73, 95% CI: 0.78–0.88.

The rebalanced data by SMOTE were used for the classification-model development. The model optimism and internal validation were evaluated by 10-fold cross-validation. The discrimination performances of models are presented in Figure 2 and Table 3. It was found that all rebalanced learning models performed with excellent discrimination. The overall discrimination performance was presented by mean AUC and 95% CI from 10-fold cross-validation. As a result, the ensemble-based (GBC) and the decision tree-based (RF) models had obtained the outperforming model discrimination with mean AUC (0.95, 95% CI 0.90 to 1.00, and 0.92, 95% CI 0.87 to 0.97), respectively. The KNN model and MLP had also achieved valid discrimination performances. Though the logistic regression had the lowest discrimination performance and low specificity (50.79%), it still provided high AUC (0.81, 95% CI 0.75 to 0.87). For the secondary metrics, a high sensitivity represents the rule-in performance (screening test), and a high specificity reflects the rule-out performance (confirm test). The models that provided outperforming sensitivity were RF (91.66%), GBC (90.4%), and Logit model (90.01%). For specificity, GBC and KNN provided excellent model specificity, which were 86.39% and 81.12%, respectively. For other alternative metrics, a positive likelihood ratio (PLR) and a negative likelihood ratio (NLR), which were not affected by data rebalancing, were also used to express a change in odds by model prediction. A high PLR means that the post-test probability of a road-traffic death is highly increased, given a positive test. Conversely, a relatively low NLP (e.g., 0.1) significantly decreases the probability of a road-traffic death, given a negative test. The best performances in both PLR and NLP were found in the GBC (6.64 and 0.11) and RF (3.68 and 0.11) models.

**Figure 3:**
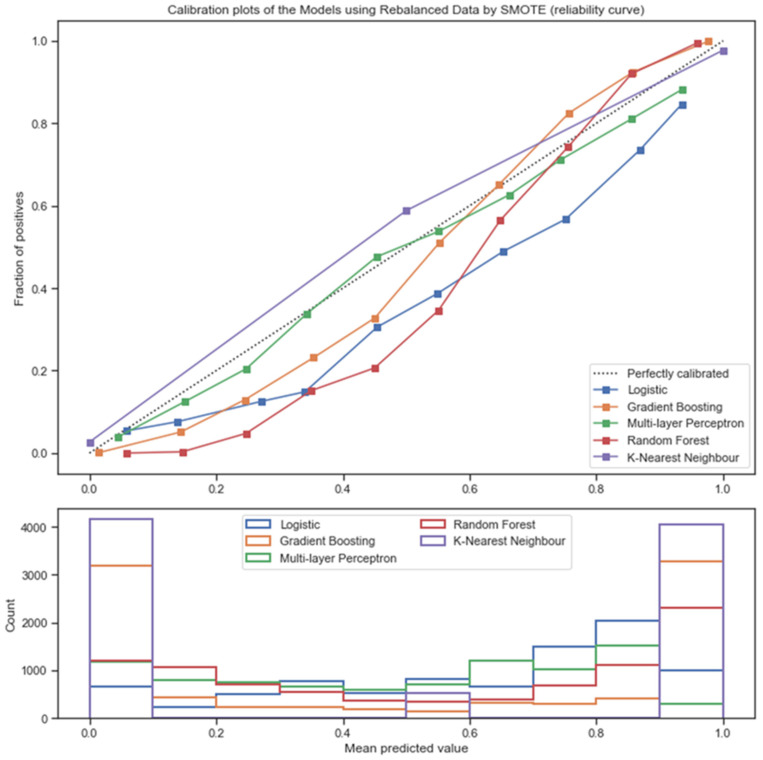
Calibration plot of the rebalanced learning classifiers.

The model calibration was visualized with the calibration plot, which compared the expected probability of road-traffic death, and the mean 10-fold cross-validation predicted the probability of each model. From Figure 3, the MLP classifier was almost perfectly calibrated, but slightly underestimated the high predicted probability. The predicted probabilities from GBC, RF, and Logistic models were underestimated in low predicted probability. However, GBC and RF appeared to be well-calibrated in high predicted probability, whereas the KKN model made a marginally overestimated predicted probability.

## 4. Discussion

Alcohol-related RTI is the most important risk factor in road-traffic mortality [4,5,6]. Currently, the alcohol limit regulations have been globally enforced for many years [1]. However, the risk of drunk driving may differ in different circumstances and contexts. It has raised the concern that only one cutoff of alcohol level might not be general enough to identify drivers at risk in all populations. As a result, the univariate predictors including BAC and others obtained poor discrimination performances. We also demonstrated that imbalanced learning in prediction-model development affected the model discrimination and calibration. Rebalanced data by the minority group oversampling using the SMOTE method significantly improved the model performances. Lastly, our study has revealed the potential of ML application in the prediction of alcohol-related road-traffic death using precrash factors combined with BAC, which can be applied as a personalized risk-prediction tool for RTI prevention in the future.

### 4.1. Limitations

Our ML models have several additional limitations. The most crucial limitation is the derived secondary data, which were cross-sectionally collected 16 years ago. Therefore, this model may be out of date and may need further updating and validation with contemporary data. Nevertheless, we decided to use these data because it was a large national survey of RTI data consisting of the alcohol testing results (BAC, BrAC). Besides, a lack of current RTI data and other data, e.g., alcohol testing results particularly for BrAC, which can be used for ML development, is an important factor. The pattern of RTI in Thailand [27], and other middle- and lower-income countries [28], has remained the same as in the past decades, and drunk driving is also the leading cause of traffic death. The ML models from our study may be helpful for these countries where the pattern of traffic accidents is similar to Thailand. Second, using the occurrence of RTI as a target outcome may be more appropriate than road-traffic mortality for the prevention. Moreover, predicted road-traffic death by our models was under the assumption that a traffic accident had occurred. Hence, the application of these models in practice should warn that these models may overestimate the risk of RTI death. The third limitation was the missing values of BAC, approximately half of all drivers were not tested. Nevertheless, we used both domain expertise and other imputation techniques to improve quality of the data. Another limitation was imbalanced data that directly impacted the model performances. A rebalancing strategy was performed to handle imbalanced data before developing the ML. We decided to use the Synthetic Minority Oversampling Technique (SMOTE) based on its effectiveness in the previous prediction model developments [29,30].

### 4.2. Interpretations

Our RTI risk models predict a mortality risk from RTI based on BAC level, driver characteristics, safety practice, and environmental factors. These precrash factors are collected by the investigating officer, e.g., driver characteristics by scanning driver license or ID card, safety practices and alcohol level at the sobriety checkpoint. The sobriety checkpoint location can be retrieved from an application programming interface requests for real-time geolocation data. The ML prediction result was the probability of road-traffic mortality (0–100%) and the classified outcomes were death (high risk) or survival (low risk) from the road-traffic accident. It should be noted that the prediction relied on the assumption that the driver had been in a traffic accident. For the probability result, the MLP model is the preferable algorithm because this model demonstrated the best model calibration (best fit between actual and predicted probability in the calibration plot) and still provided good model discrimination. In contrast, the boosting-based (GBC) and ensemble-based (RF) models are our suggested methods for classifying the driver into high risk and low risk of road-traffic death. According to their ground theorems, based on an extension of the decision-tree method, these models tend to be better at predicting the binary outcome. The officers can use the prediction result to communicate with the drunk driver, particularly those whose alcohol level is under legal limitation. BrAC values may be feasible for use in the models according to an impractical BAC measurement.

### 4.3. Implications

Our study demonstrated that the ML algorithm using precrash predictors and BAC can precisely predict the road-traffic mortality risk of the drunk driver. We also showed that using only legal cutoff or BAC levels might provide very poor discrimination performance for the driver at risk. In addition, an alcohol level under the legal threshold might cause an impairment based on various precrash conditions or driver characteristics [14,15,16]. These models can be applied as personalized risk identification and an alternative personalized legal limit. The officer at the checkpoint can use the prediction result to raise the awareness of the drunk driver. Since many countries have had RTI data collection and report systems for years, it is possible to integrate these ML models with RTI data management systems as an innovative RTI prevention strategy. Furthermore, BrAC may be the preferred predictor method as it is simpler to implement at screening sites than BAC measurements, especially if used as a screening strategy. However, we assumed that BAC values are relatively similar to BrAC measurements with some variation. BrAC has been widely accepted as the standard alcohol measurement. Thus, it is possible to apply BrAC level instead of BAC data. The advancement of government data integration and exchange will increase several possibilities to utilize the data for other public health prevention tasks [31]. For instance, the added predictors, e.g., driving experience, underlying health conditions, or the driving route conditions, which were already collected by the government, may improve the performance of the updated ML model in the future.

## 5. Conclusions

Our study developed novel machine-learning algorithms with internal validation to identify model performances using the standard alcohol level measurement combined with simple precrash factors. Our machine-learning models can predict road-traffic mortality risk with a good model discrimination and calibration. Nonetheless, model updating and external validation with current data are required to ensure the possibility of model implementation in the future.

## Figures and Tables

**Table 1:** Baseline driver characteristics divided by the target outcome.

Characteristics	Total (*n* = 4794)				*p*-Value
	Death (*n* = 429)		Survive (*n* = 4365)		
	*n*	%	*n*	%	
Age, median (IQR), years	26	(19)	30	(6)	<0.001 *
Gender					
Male	393	91.61	3804	87.15	0.008
Female	36	8.39	561	12.85	
Alcohol					
BAC level, median (IQR), mg%	15	156.70	1	130.00	0.051 *
Alcohol odor on breath	321	74.83	2915	66.78	<0.001
Type of vehicle					
Bicycle	20	4.66	133	3.05	0.069
Motorcycle	378	88.11	3978	91.38	0.038
4-wheel car	26	6.06	210	4.81	0.254
Commercial truck, semitrailer, and trailer	5	1.17	37	0.85	0.500
Safety belt used ^a^	(*n* = 31)		(*n* = 247)		
Yes	3	9.68	79	31.98	0.010
No	28	90.32	168	68.02	
Helmet used ^b^	(*n* = 398)		(*n* = 4111)		
Yes	40	10.05	709	17.25	<0.001
No	358	89.95	3402	82.75	
Place of accident					
Urban	50	11.66	822	18.83	<0.001
Suburban	148	34.50	1480	33.91	0.805
Rural	231	53.85	2063	47.26	0.009
Driving across provinces	111	25.87	587	13.45	<0.001
Time of accident					
8:01 a.m. to 4:00 p.m.	106	24.71	1208	27.67	0.189
4:01 p.m. to 12:00 a.m.	97	22.61	1198	27.45	0.031
12:01 a.m. to 8:00 a.m.	226	52.68	1959	44.88	0.002

^a^ only 4-wheel car, commercial truck, semitrailer and trailer driver; ^b^ only motorcyclist and bicyclist; BAC: Blood alcohol concentration. All *p*-values of the categorical variables were obtained from chi-squared test; For the continuous variables, *p*-values were obtained from * Rank-sum test (nonparametric).

**Table 2:** The association between candidate predictors and death from road-traffic injury.

Characteristic	OR	*p*-Value	aOR	*p*-Value	AUC	95% CI
Age, years (median, IQR)	1.01	<0.001	1.01	<0.001	0.56	0.53–0.58
Male	1.60	0.008	1.42	0.059	0.52	0.51–0.54
BAC level, mg% (median, IQR)	1.00	0.051	1.00	0.052	0.53	0.49–0.55
Motorcycle	0.72	0.038	0.74	0.071	0.48	0.46–0.50
Safety belt used	0.38	0.010	0.18	0.005	0.49	0.49–0.50
Helmet used	0.53	<0.001	0.55	<0.001	0.46	0.45–0.48
Place of accident: Suburban	1.03	0.805	1.57	0.008	0.50	0.48–0.53
Place of accident: Rural	1.30	0.009	1.74	0.001	0.53	0.51–0.55
Driving across provinces	2.25	<0.001	2.12	<0.001	0.56	0.54–0.58
Driving at night (12:01 a.m. to 8:00 a.m.)	1.37	0.002	1.25	0.035	0.53	0.51–0.56

aOR: Adjusted odd ratio from the multivariable logistic regression model; AUC: Area under the received operating characteristic curve; OR: Odd ratio from univariable analysis.

**Table 3:** The Discrimination Performance of Mortality Prediction Models with Rebalanced data using SMOTE.

Models	Model Prediction	(Death/ Survival)	AUC		Likelihood Ratio		Sensitivity	Specificity
			Mean	95% CI	Positive	Negative		
GBC	Death	(3946/594)	0.95	0.90–1.00	6.64	0.11	90.4	86.39
	Survival	(419/3771)						
RF	Death	(4001/1086)	0.92	0.87–0.97	3.68	0.11	91.66	75.12
	Survival	(364/3279)						
MLP	Death	(3462/1299)	0.83	0.78–0.88	2.67	0.29	79.31	70.24
	Survival	(903/3066)						
Logit	Death	(3929/2148)	0.81	0.75–0.87	1.83	0.2	90.01	50.79
	Survival	(436/2217)						
KNN	Death	(3573/824)	0.86	0.83–0.89	4.34	0.22	81.86	81.12
	Survival	(792/3541)						

AUC, Area under the received operating characteristic curve; GBC, Gradient Boosting classifier; KNN, K-Nearest Neighbor; Logit, Logistic regression; MLP, Multilayer Perceptrons; RF, Random Forest.

## Data Availability

The data presented in this study are available on request from the corresponding author.

## Supplementary Materials

The following are available online at www.mdpi.com/article/10.3390/ijerph181910540/s1, Table S1: The parameter setting of the derived models, Table S2: The discrimination performance of mortality prediction models using imbalance data, Figure S1: Receiver Operating Characteristic curves from 10-fold cross-validation of the imbalanced learning classifiers, Figure S2: Calibration plot of the imbalanced learning classifiers.
