# Supplementary material for: Mortality-Risk Prediction Model from Road-Traffic Injury in Drunk Drivers: Machine Learning Approach
Source: Int J Environ Res Public Health. 2021 Oct 8;18(19):10540. doi: 10.3390/ijerph181910540 (PMC8508427; doi:10.3390/ijerph181910540)

Supplementary Materials

Table S1. The parameter setting of the derived models

Table S2. The discrimination performance of mortality prediction models using imbalance data

Figure S1. Receiver Operating Characteristic curves from 10-fold cross-validation of the imbalanced learning classifiers

Figure S2. Calibration plot of the imbalanced learning classifiers

This supplementary material has been provided by the authors to give readers additional information about their work.

Table S1. The parameter setting of the derived models.

Type of Model	Parameters settings
Imbalanced learning	
GBC	{'criterion': 'friedman_mse', 'learning_rate': 0.1, 'loss': 'deviance', 'max_depth': 4, 'max_features': 'sqrt', 'min_impurity_decrease': 0.1, 'n_estimators': 10}
RF	{'bootstrap': True, 'class_weight': {0: 1, 1: 2}, 'max_depth': 90, 'max_features': 'sqrt', 'min_samples_leaf': 7, 'min_samples_split': 8, 'n_estimators': 200}
MLP	{'activation': 'relu', 'alpha': 0.001, 'early_stopping': False, 'hidden_layer_sizes': 26, 'learning_rate': 'adaptive', 'learning_rate_init': 0.005, 'max_iter': 10000, 'power_t': 0.5, 'solver': 'adam', 'warm_start': False}
Logistic	{'C': 0.0001, 'class_weight': {0: 1, 1: 2}, 'penalty': 'l1', 'solver': 'saga'}
KNN	{'algorithm': 'brute', 'metric': 'manhattan', 'n_neighbors': 11, 'weights': 'uniform'}
SMOTE	
GBC	{'criterion': 'friedman_mse', 'learning_rate': 0.1, 'loss': 'exponential', 'max_depth': 10, 'max_features': 'auto', 'min_impurity_decrease': 0.1, 'n_estimators': 100}
RF	{'bootstrap': True, 'class_weight': {0: 1, 1: 2}, 'max_depth': 80, 'max_features': 'auto', 'min_samples_leaf': 3, 'min_samples_split': 8, 'n_estimators': 300}
MLP	{'activation': 'logistic', 'alpha': 0.0001, 'early_stopping': False, 'hidden_layer_sizes': 93, 'learning_rate': 'constant', 'learning_rate_init': 0.005, 'max_iter': 10000, 'power_t': 0.5, 'solver': 'adam', 'warm_start': False}
Logistic	{'C': 1.623776739188721, 'class_weight': {0: 1, 1: 2}, 'penalty': 'l1', 'solver': 'liblinear'}
KNN	{'algorithm': 'ball_tree', 'metric': 'manhattan', 'n_neighbors': 2, 'weights': 'distance'}

GBC, Gradient Boosting classifier; KNN, K-Nearest Neighbor; Logit, Logistic regression; MLP, Multi-layer Perceptrons; RF, Random Forest.

Table S2. The discrimination performance of mortality prediction models using imbalance data.

Model	AUC		Likelihood ratio		Sensitivity	Specificity	Predictive value	
	mean	95% CI	Positive	Negative			Positive	Negative
GBC	0.62	0.58 - 0.67	1.77	1.00	0.31	99.83	14.29	91.41
RF	0.60	0.56 - 0.65	1.44	0.90	26.34	81.76	12.43	91.87
MLP	0.61	0.56 - 0.65	1.62	0.98	4.89	96.98	13.22	91.55
Logistic	0.63	0.57 - 0.68	3.39	1.00	0.23	99.93	25.00	91.06
KNN	0.54	0.51 - 0.56	10.62	1.00	0.31	99.97	50.00	91.42

AUC: Area under the received operating characteristic curve, GBC, Gradient Boosting classifier; KNN, K-Nearest Neighbor; Logit, Logistic regression; MLP, Multi-layer Perceptrons; RF, Random Forest.

Figure S1. Receiver Operating Characteristic curves from 10-fold cross-validation of the imbalanced learning classifiers.

1a. Gradient Boosting Classifier (GBC) model

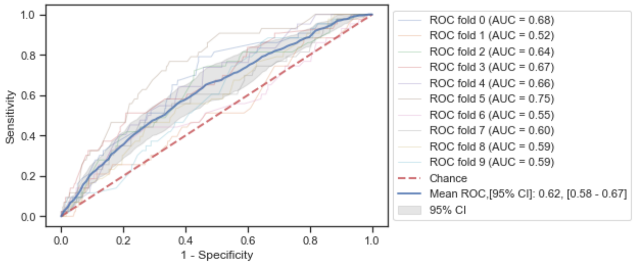

1b. Random Forest (RF) model

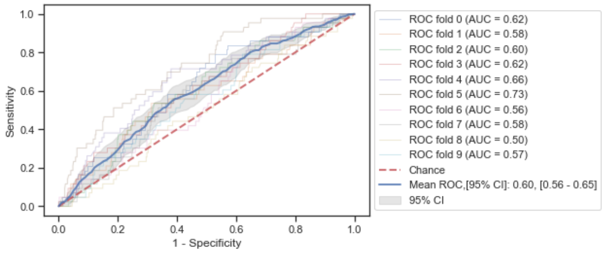

1c. Multi-Layer Perceptron (MLP) model

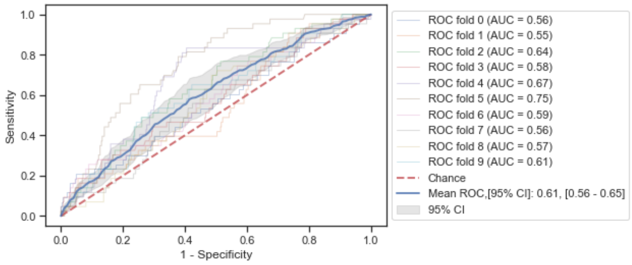

1d. Regularized Logistic Regression (Logit) model

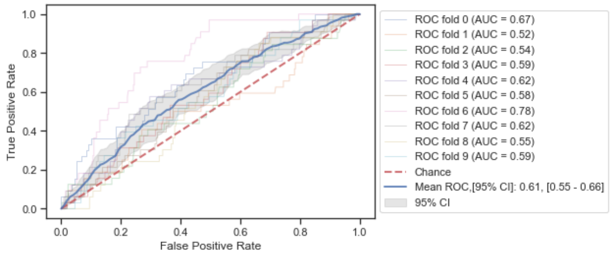

1e. K-Nearest Neighbor (KNN) model

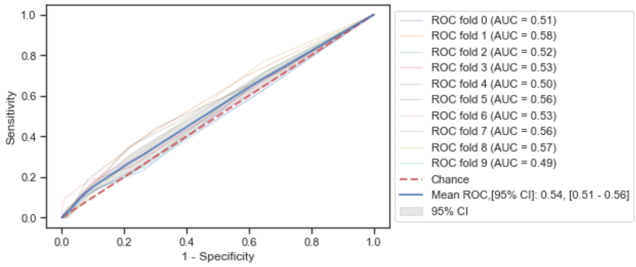

ROC, Received operating characteristic curve

Figure S2. Calibration plot of the imbalanced learning classifiers.

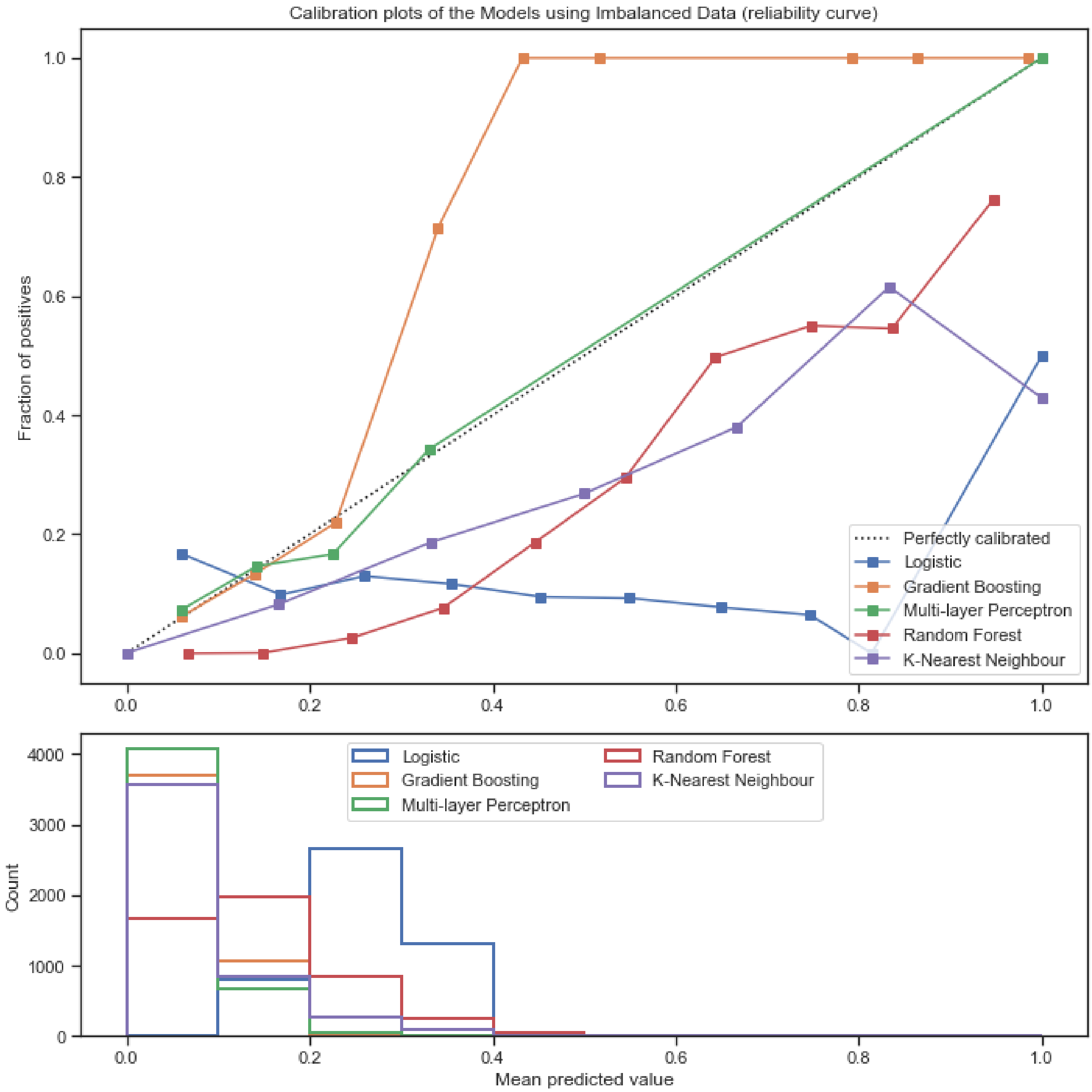

Supplement: Supplementary file 1 [file ijerph-18-10540-s001.zip › ijerph-1387522-supplementary.pdf]
